# Dual vs. Single Tasking During Circular Walking: What Better Reflects Progression in Parkinson's Disease?

**DOI:** 10.3389/fneur.2019.00372

**Published:** 2019-05-14

**Authors:** M. Encarna Micó-Amigo, Idsart Kingma, Sebastian Heinzel, Susanne Nussbaum, Tanja Heger, Rob C. van Lummel, Daniela Berg, Walter Maetzler, Jaap H. van Dieën

**Affiliations:** ^1^Department of Human Movement Sciences, Vrije Universiteit Amsterdam, Amsterdam Movement Sciences, Amsterdam, Netherlands; ^2^Department of Neurology, Christian-Albrechts-University, Kiel, Germany; ^3^Department of Neurodegeneration, Center of Neurology, Hertie Institute for Clinical Brain Research, University of Tübingen, Tübingen, Germany; ^4^DZNE, German Center for Neurodegenerative Diseases, Tübingen, Germany; ^5^McRoberts B.V., The Hague, Netherlands

**Keywords:** walking, accelerometry, movement disorders, gait analysis, Parkinson's disease, dual-tasking-interferences, body-fixed-sensors, cognition

## Abstract

**Background and Aim:** Reliable, valid and sensitive measures of dual-task-associated impairments in patients with Parkinson's disease (PD) may reveal progressive deficits unnoticed under single-task walking. The aim of this study was to quantitatively identify markers of progressive gait deficits in idiopathic PD while walking over a circular trajectory condition in single-task walking and in different dual-task conditions: (1) circular walking while checking boxes on a paper sheet as fast as possible and (2) circular walking while performing subtraction of 7 as fast as possible. In addition, we aimed to study the added value of dual-tasking assessment over single (circular) walking task assessment in the study of PD progression.

**Methods:** The assessments were performed every 6 months over a (up to) 5 years period for 22 patients in early-stage PD, 27 patients in middle-stage PD and 25 healthy controls (HC). Longitudinal changes of 27 gait features extracted from accelerometry were compared between PD groups and HCs using generalized estimating equations analysis, accounting for gait speed, age, and levodopa medication state confounders when required. In addition, dual-task-interference with gait and cognitive performance was assessed, as well as their combination.

**Results:** The results support the validity and robustness of some of the gait features already identified in our previous work as progression markers of the disease in single-task circular walking. However, fewer gait features from dual-task than from single-task assessments were identified as markers of progression in PD. Moreover, we did not clearly identify progressive worsening of dual-task-interference in patients with PD, although some group differences between early and middle stages of PD vs. the control group were observed for dual-task interference with the gait task and with the concurrent tasks.

**Conclusions:** Overall, the results showed that dual-tasking did not have added value in the study of PD progression from circular gait assessments. Our analyses suggest that, while single-task walking might be sensitive enough, dual-tasking may introduce additional (error) variance to the data and may represent complex composite measures of cognitive and motor performance.

## Introduction

Circular gait is a challenging locomotor task which involves complex cognitive-motor control (1). Turning requires commands from the central nervous system to integrate inter-segmental coordination, to control axial rotation and to orient gaze toward the intended trajectory, while maintaining dynamic balance stability (2–4). In this complex process, cognitive resources such as attention (5), visuospatial function and executive function are involved (1, 3). The simultaneous execution of gait with an additional (motor and/or cognitive) task involves sharing these cognitive resources (capacity-sharing model) and/or switching (bottleneck model) the attention between tasks (6, 7). This leads to a delayed or worse performance of one or both tasks, relative to single-tasking (8, 9). When considering the additional demands of circular gait, such effects of dual tasking might be more pronounced in circular compared to straight-line gait.

Cardinal motor symptoms of Parkinson's disease (PD): rigidity, tremor, postural instability and bradykinesia, can contribute to both gait and dual-task deficits (10) and increase with disease progression (11). The loss of automaticity in patients with PD (12) challenges the control of gait and might increase reliance on cognitive resources to optimize the motor control (7, 13). This limits the availability of cognitive resources in PD to perform a second task while walking (1, 7, 8, 13). Also, cognitive impairments in PD, which are related to basal ganglia dysfunction (14), are often progressive and could involve increasing limitations on working memory and learning processes (15), response inhibition, deficits or inflexibility in dividing/alternating attention (16), problems in planning, and impaired executive and visuospatial functions (17–19), which may all be relevant to dual-task walking (1). Consequently, individuals with PD may be particularly and progressively susceptible to dual-task interference (8), especially when walking over a trajectory which involves turning (3).

As a consequence of limited available attention, patients with PD might, in dual-task conditions, prioritize one task over the other [i.e., focus more attention on the performance of one of the two tasks (20)] depending on fatigability and psychological factors, such as motivation, anxiety, balance confidence, perceived importance of task, on motor and cognitive abilities and on environmental/situational factors (8). However, under dual-tasking conditions that include walking, patients with PD generally divide their attention among both tasks at the expense of gait performance (posture second strategy) (21). This could lead to reduced gait speed and stride length, increased gait variability, gait asymmetry and impaired bilateral coordination, even in optimally treated patients (22); potentially compromising their safety and increasing their risk for falling (1, 10, 21, 23) under usual dual-tasking conditions in daily-life (10, 21). Moreover, the greater presence of mobility deficits in PD seems associated to a higher sensitivity to dual-tasking interferences on gait performance (22), which suggests that the assessment of gait under dual-tasking might reveal more gait deficits in patients with PD than under single walking task conditions. Prioritization strategies during the performance of dual-tasking protocols may be influenced by walking situations (straight or curved walking path), with a worse gait performance under a curved trajectory, which is often present in daily-life (24). This highlights the importance of identifying dual-tasks impairments under circular walking in the assessment of PD (25). Specifically, well-designed dual-task assessments and interventions may create awareness about dual-task-related limitations and associated risks (7, 13).

In our previous study (26), we identified progression markers of PD (i.e., quantitative gait performance indicators of the decline with time in PD relative to control subjects) by the assessment of single-task walking. However, progressive deficits (reflected by worsening of gait features) might have been unnoticed under single-task circular walking. Evaluation of dual-tasking protocols may reveal the development of compensation strategies which preserve gait functioning when the basal ganglia are dysfunctional (27). Moreover, since reliable, valid and sensitive measures of worsening of impairments associated with dual-tasking in PD are still lacking (10), longitudinal studies characterizing the evolution of the cognitive and locomotor profile of PD not only under single, but also under dual-tasking conditions are required.

Accordingly, the aim of this study was to quantitatively identify markers of progressive gait deficits in idiopathic PD while walking a circular trajectory under a single-task condition and two dual-task conditions: (1) circular walking while marking crosses on a paper sheet and (2) circular walking while subtracting series of 7 (6). We investigated the added value of dual-task over single-task assessment by analyzing the effects on gait performance, as well as the interference of gait with each of the concurrent tasks. We hypothesized that more gait features would be identified as progression markers of PD in dual-task than in single-task walking and we expected to identify progression of dual-task-interference throughout the course of the disease in patients with PD.

## Materials and Methods

### Participants

As part of the prospective observational MODEP study (Modeling epidemiological data to study PD progression), assessments were performed every 6 months over a 5-years period in 74 participants, 49 patients diagnosed with idiopathic PD and 25 healthy controls (HC). All participants were recruited from the outpatient clinic of the Department of Neurodegeneration, Center of Neurology, University Hospital of Tübingen, Germany.

The Declaration of Helsinki was respected; local ethics committee approval was obtained (Medical Faculty, University Hospital of Tübingen, No. 46/2010 BO1) and all subjects provided informed written consent for participation in the study and for publication of individual, anonymized data.

The participants were selected according to the following inclusion criteria: (a) age between 40 and 85 years; (b) stable medication for 2 weeks prior to inclusion; (c) absence of cognitive impairment based on a minimum score of 25 points in the Mini Mental State Examination (MMSE) (28). All participants underwent a clinical assessment which included: medical history, medication intake and neurological examination. The participants of the PD group were diagnosed with idiopathic PD according to the United Kingdom Brain Bank Society criteria (29) and did not present any other neurological disorder, nor dysexecutive syndrome. The participants of the control group had no neurological disease.

A priori classification of the PD group was performed, and each group was compared to the reference group (HC) in order to study motor symptom progression at different stages of the disease. Analogous to previous work (30), a classification, based on the time since clinical PD diagnosis, stratified patients into early-stage PD (Early-PD; < 4 years, *N* = 22) and middle-stage PD (Mid-PD; ≥ 4 years, *N* = 27). An overview of demographic and clinical data is presented in Table 1.

**Table 1:** Demographics and clinical data of participants at baseline (first visit).

		**PD–Early (*N* = 22)**	**PD–Mid (*N* = 27)**	**HC (*N* = 25)**
Demographics	Gender (female) [%]	9 (40.9)	10 (37.0)	10 (40.0)
	Age [years]	61.2 [41–73]	65.3 [43–76]	63.6 [50–75]
	Total height [m]	1.74 ± 0.10	1.73 ± 0.08	1.71 ± 0.08
	Mass [kg]	81.0 ± 19.1	78.5 ± 13.0	76.0 ± 11.3
	BMI [kg/m^2^]	26.7 ± 5.2	26.2 ± 3.7	25.9 ± 3.2
	MMSE (1–30)	28.3 [26-30]	28.4 [25–30]	28.9 [26–30]
	Academic Education [years]	10.5 [9–13]	11.0 [9–13]	11.0 [9–13]
	Disease duration [years since diagnose]	1.2 [0–3] M	7.1 [4-11] E	
	Disease duration [years since first symptoms]	2.0 [0–4] M	8.0 [5–11] E	
	Age at diagnose [years]	60.0 [40–72]	58.2 [35–71]	
	Age at manifestation [years]	59.2 [39–70]	57.3 [35–71]	
Clinical	Hoehn & Yahr (0–4)	1.7 [1–3] M	2.4 [1–4] E	
	MDS–UPDRS III (0–132)	21.5 [5–32] H,M	35.8 [8–68] H, E	1 [0–7]
	Number of PIGD–Number of TD	7–12	15–12	
	Tremor subscore of UPDRS III (0–44)	24.8 ± 8.9 M	40.9 ± 14.1 E	
	Gait subscore of UPDRS III (0–20)	1.1 [0–3] H,M	3.7 [0–14] H,E	0.0 [0.0–1.0]
	Daily levodopa medication equivalent dose [mg]	229.7 [0–607] M	689.3 [80–1300] E	

*Data are presented as mean ± standard deviation for the parametric data and median [range] for the non-parametric data. In case of gender, data are presented as a number of females and (the percentage of females over the total number of participants for each group). Significant differences (p < 0.05) with respect HC are marked with a subscript “H”, whereas significant differences with respect Early-PD are marked with a subscript “E”, and with respect to Mid-PD with a subscript “M.” Early-PD, patients at early stages of Parkinson's disease; Mid-PD, patients at a mid or advanced stages of Parkinson's Disease; HC, healthy controls; BMI, body-mass-index; MMSE, Mini-Mental-State-Examination score; MDS – UPDRS III, motor section of Unified Parkinson's disease rating scale as defined by the Movement Disorders Society; PIGD, patients with predominant postural instability and gait disorder clinical phenotype; TD, patients with tremor dominant clinical phenotype (31). The levodopa equivalent dose was calculated as recommended by Tomlinson et al. (32)*.

Visits differed with respect to medication state within and between-subjects. Sixteen percent of the assessments were in ON state. The medication ON condition was defined as a time period of 30 min to 3 h after the intake of the usual dose of dopaminergic medication (prescribed by the neurologist for an optimal medical treatment) and considering each participant's perception of having a “Good On Phase.” Treatment induced dyskinesia was uncommon in the PD group (six subjects presented dyskinesia at a single visit, one subject at two visits and three subjects at three visits). These patients presented dyskinetic movements after 7.9 years (in average) of diagnosis. At baseline, twelve patients presented motor fluctuations induced by medication, eleven of them with mid-stage PD and one with early stage PD. Six patients in middle-stage PD presented freezing of gait symptom. Four participants were rated with a score of 1 out of 4 in the freezing of gait section of the Unified Parkinson's Disease Rating Scale (UPDRS) (31), while two participants were rated with a score of 3 out of 4.

### Protocol

**Figure 1:**
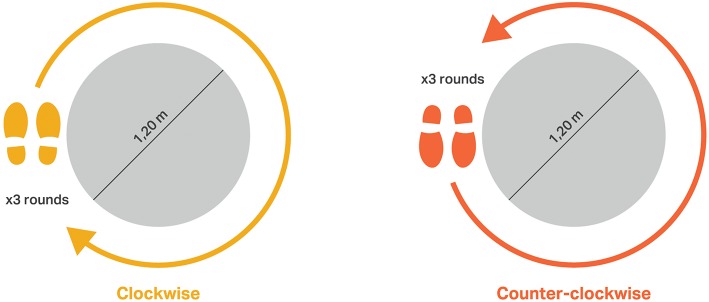
Schema of the task “walking in circles”: **(left)** clock-wise, **(right)** counter-clock-wise.

Three different single tests were performed as previously proposed (25):

1. **Walking in circles** (“Walk”), in which the subjects, wearing their own shoes and a sensor on the lower back (see below), were asked to walk 3 rounds around a circle of 1.2 meter diameter at their preferred speed. The trial started after a verbal countdown and ended when the subjects completed the third round and reached their original position. This test was performed clock-wise and counter-clock-wise (Figures 1A,B).
2. **Marking crosses** (“Mark”), in which the participants held a clipboard in their non-dominant hand and a pen in the other hand. They were requested to mark with a cross, as fast as possible, each of the 32 boxes drawn on a sheet of paper. The instruction was as follows: “Please mark each of the boxes on the sheet of paper with a cross, as fast as you can.” There was no instruction about where to start and end crossing boxes, neither about the order of crossing.
3. **Subtracting** (“Subtract”), in which the participants had to subtract, as fast as possible, serial 7 digits from a randomly chosen three-digit number until completing 10 subtractions. The participants had to verbally indicate the resulting numbers. The instruction was as follows: “Please subtract serial 7 digits as fast as you can from the number I will shortly tell you, until I will interrupt you.”

The number of checked boxes, number of subtractions and number of subtraction errors were recorded for each trial. Performances of Mark and Subtract tasks were assessed, respectively, as the total number of checked boxes/total duration to perform the task [boxes/s], and the total number of correct subtracting series/total duration to perform the task [subtractions/s].

Subsequently, two dual-tasks were performed, for which no hint on task prioritization was given [to omit an external influence on the prioritization process (20)]:

**(1** **+** **2) Walking in circles while marking crosses** (“W-Mark”). In this case, the subjects were asked to mark as many boxes as possible (without limit in the amount of boxes to check), while walking three rounds around the circle. The “Mark” task involves motor tasks (using the upper limbs for holding the paper and marking the boxes), which performed simultaneously with the walking condition requires the division of attention (6, 25). Moreover, this task requires the interruption of the gaze over the circular trajectory and over the feet to visually focus on the paper, which challenges the performance of circular walking (33).

1. **(1** **+** **3) Walking in circles while subtracting series** (“W-Subtract”). In this case, the subjects were asked to verbally report as many subtractions as possible, while walking three rounds around the circle. The “Subtract” task is an executive task which involves highly challenging cognitive flexibility (arithmetic skills), and in combination with circular gait requires division of attention (6, 25). Circular gait combined with these tasks could further worsen the gait performance of patients with PD in view of the asymmetric pattern than under single walking (22).

The participants were asked to perform both tasks simultaneously. No particular instructions were given to the participants regarding which task to prioritize, nor regarding which task to start first. Both dual-tasking protocols were performed clock-wise and counter-clock-wise and were alternated to avoid the effects of direction.

### Instrumentation

The measurement system consisted of a BFS (DynaPort® Hybrid, McRoberts), a remote control and a portable computer on which the DynaPort software was installed. The sensor consists of a triaxial accelerometer and a triaxial gyroscope, and stores data at a rate of 100 samples per second. The accelerometer is a DC type sensor and therefore it is also sensitive to gravity. It has a range of ± 19.62 m/s^2^ and a resolution of 0.00981 m/s^2^. The sensor was inserted in an elastic belt, placed around the waist so that the sensor was positioned at the level of the lowest lumbar vertebra (L5). All protocol tests were implemented on a computer. Dedicated software was activated with a remote control to initiate and stop data collection.

### Gait Assessment

A comprehensive set of 24 gait characteristics was estimated from triaxial acceleration signals to assess gait during single and dual-tasking tests. This set included the following gait features: number of steps; total duration; asymmetry of step time; median and variability of stride time; standard-deviation (SD) of accelerometry in each direction: vertical (VT), medio-lateral (ML) and anterior-posterior (AP) (34, 35); step and stride regularity, estimated for vertical VT and AP accelerations (36); harmonic ratios calculated for each direction (35, 37, 38), which reflect step-to-step symmetry (39, 40); indices of harmonicity calculated for each direction, reflecting gait smoothness (41, 42); normalized peak powers calculated for each direction as the magnitude of the power spectral density (PSD) at the dominant peak, normalized by the total integrated PSD (43), reflecting the periodicity of the signal; and the width of peak powers, estimated as the width of the PSD at the dominant frequency (43), reflecting gait consistency or variability of dominant cycles in the signals, i.e., steps in VT and AP accelerations and strides in ML accelerations.

The average of gait features extracted from both trials (clock-wise and counter-clock-wise direction) for each protocol was obtained and used for further analysis. Details on the calculation of the gait parameters were reported in our previous publication (26), concerning circular gait assessments of low-back accelerometry under non dual-task condition.

In our previous work (26) we found associations (in the expected direction) of most of the proposed gait features (assessed under single walking conditions) with the summed score of the items concerning gait assessment from the motor section of the Unified Parkinson's Disease Rating Scale (31). This suggests substantial construct validity of the proposed gait features.

### Dual-Task Interference

Dual-task interference “DTI” assesses trade-offs between concurrent task performances (10) and was calculated as: DTI (%) = [(dual-task feature—single task feature)/single task feature]*100 (8). This formula gives information about the percentage change relative to the single-task value. These were individually calculated for both Mark and Subtract performances and for each gait feature that presented a significant effect (*p* < 0.05) or a trend toward significance (*p* < 0.1) for the interaction group x time in at least one of the gait tests (Walk, W-Mark, or W-Subtract). This interaction was chosen as a criterion because progressive changes over time on dual-task interferences are expected to be different between groups. Thus, only gait features that presented progressive worsening in PD are considered, either extracted from single task or from dual task conditions.

The assessments of DTI on the gait performance as well as on the other tasks (“Mark” and “Subtract”) are important to consider since dual-tasking could lead to: (a) a reciprocal dual-task decline in both tasks (mutual interference), (b) no change on the Mark or Subtract tasks relative to single tasking (i.e., only gait interference), or (c) improvement on Mark or Subtract tasks relative to single tasking (i.e., gait interference with benefit on the Mark/Subtract tasks) due to additional attention (prioritization). Thus, the calculation of DTI on each task is required to understand potential task prioritization (8). As participants might show task prioritization (8), we also calculated, within each dual task, the sum of the DTI of the two tasks, i.e., “S-DTI” = DTI % (gait feature interference under dual-tasking) + DTI % (Mark/Subtract interference under dual-tasking)” to obtain a measure of dual-task interference independent of task prioritization (6, 25), i.e., considering potential mutual interferences simultaneously, rather than solely possible interferences on a particular task. For features in which a higher value indicates a worse gait performance (e.g., duration of trial), a positive DTI% indicates a reduced performance under dual-tasking relative to single walking task. Thus, for these features, the calculation of S-DTI required multiplying the DTI% by −1, before summing it to the DTI% on subtracting or marking crosses. In this way, negative values on S-DTI% would indicate a “reduced performance” or an overall cost when dual-tasking compared to single tasking, and the opposite for positive values.

### Statistical Analysis

Gait features and Mark/Subtract performances assessed from biannual visits were averaged to obtain annual data, i.e., year 1 (baseline) represents the average of visit 1 and visit 2 data, year 2 (visits 3–4), year 3 (visits 5–6), year 4 (visits 7–8), and year 5 (visits 9–10). In case of single missed visits, the remaining value of that year was taken as the average. Averaging within years was done based on the assumption that it will reduce some of the variance caused by day-to-day differences as well as measurement errors, thereby increasing the power to detect true effects.

The longitudinal data (gait features, Mark/Subtract performances and DTIs) of this prospective study were statistically analyzed with generalized estimating equations (GEE) (44), using identity-link functions with normal distributions. Logarithmic transformation was applied to improve distribution of skewed data (45). In case of negative data, an offset value (absolute 110% of the minimum) was added to guarantee positive data prior to logarithmic transformation. In addition, all features and DTIs were scaled to obtain zero means and unit variances (z-scores transformation) (46) to allow easy comparison of regression coefficients (β-values) between the proposed variables. We used the between-subject variance over the combined groups for this normalization.

Inspection of correlations between measurement points confirmed the assumption of an equicorrelated data structure. Gait features, Mark/Subtract performances or DTIs of interest were the dependent variables. The models comprised the factors Group (coded with 0 = HC and 1 = Early-PD or Mid-PD), Time (number of year, with baseline set to 0) and the Interaction Group x Time. In addition, the effect of potential confounders was analyzed by including the following factors: Total duration of trial (to assess the effect of gait speed), Age and ON/OFF medication stage (0 = OFF, 1 = ON; 0.5 if one of two visits of 1 year was in ON). ON medication was present in 16.4% of the visits of patients with PD. The HC did not take any anti-Parkinsonian medication, therefore, for this group the score was always 0.

For each of the gait features obtained from the “Walk” protocol, we included all potential confounders. We identified the confounder with the highest *p*-value, and this was removed if not significant. Then, the analysis was repeated with the remaining factors until only significant confounders were kept in the analysis. For the gait features obtained from “W-Mark” and “W-Subtract,” if Age and/or ON-OFF medication stage were significant in the analysis of the corresponding gait feature from the “Walk” protocol, these were kept in the analysis. Subsequently, we evaluated the additional effect of Total duration of trial as confounder, and if significant, this was also kept in the analysis. For the analysis of Mark/Subtract performances, DTIs on these performances and for the feature Total duration of trial, only Age and ON/OFF medication were considered as confounders and included if significant. For the analysis of DTIs and S-DTI of gait features, the corresponding DTI in Total duration of trial was considered in addition to Age and ON/OFF medication stage as potential confounder. Only significant confounders were included.

We report *p*-values, β-values and standard errors (SE) obtained from the GEE for time (reported in the Supplementary Material), group and their interaction. The groups were the predefined PD groups (Early-PD or Mid-PD) vs. the reference group (HC) for each gait feature, for Mark/Subtract performances and for DTI. In addition, we report the confounders which were significant and therefore accounted for in the analysis (the confounders for gait features and for Mark/Subtract performances are reported in the Supplementary Material). The level of significance of the GEE models was set to α = 0.05 (two-sided). We did not correct the *p*-values for multiple comparisons, as this is an explorative study and we were concerned about possible Type II errors.

We also reported the percentage of annual change with respect to the first year for the features or DTIs that showed a trend toward significance (*p* < 0.1) in the interaction group x time. This was calculated separately for each group by applying the GEE analysis on non-transformed data, including the factor Time (reported in the Supplementary Material) and the significant confounders. The resulting β-value and standard error of the time factor were divided by the mean value of data concerning the first year of all subjects within the group. All results in the expected direction were marked with green color and with red for the unexpected directions. All calculations were performed with a custom Matlab program (Natwick, Massachusetts: The MathWorks Inc., R2016a).

### Potential Markers of Progression in Parkinson's Disease

As proposed in our previous study (26), we considered several criteria to identify progression markers in different stages of PD.

For markers of early-stage PD, these features should show:

E1) a Group (Early-PD vs. HC) x Time interaction in the expected direction, reflecting faster decline of gait quality in the Early-PD compared to HC.
E2) a significant percentage of annual change in the Early-PD group, in the expected direction.
E3) a significant effect of Group (Mid-PD vs HC) in the expected direction, reflecting impaired gait performance of the Mid-PD with respect to HCs.

The third criterion (E3) implies that the gait symptoms that worsen (i.e., their severity increases with time) in early stages PD are expected to still be present at more advanced stages of the disease, such as middle-stages PD. Thus, these gait symptoms remain at baseline in middle stages of the disease.

For markers of middle-stages PD the criteria are:

M1) a significant Group (Mid-PD vs. HC) x Time interaction in the expected direction, reflecting faster worsening of gait quality in the Mid-PD compared to HCs.
M2) a significant percentage of annual change in the Mid-PD group, in the expected direction.

Since no data on advanced PD were available, no criterion analogous to criterion 3 for Early-PD could be defined for the Mid-PD group. In view of the use of multiple criteria, we accepted single criteria to be met with α = 0.10. Assuming independence of criteria, this implies α-levels of 0.0001 for the definition or progression markers in the Early-PD and 0.001 for the Mid-PD. These α-levels are conservative compared to Bonferroni correction, which for the 24 gait features would yield an α-level of 0.002. We additionally report differences between Early-PD and Mid-PD vs. HC, for which we used a conventional α-level of 0.05.

The potential added value of dual-tasking assessment in the study of progression of PD should be reflected in consistent progressive changes of dual-tasking-interferences (DTI and S-DTI) with the proposed gait features. To explore that, the previous criteria were considered for DTI and S-DTI of the proposed gait features.

## Results

### Descriptive Statistics

Demographic variables did not significantly differ between the HC and the Early-PD groups, nor between the HC and Mid-PD groups (Table 1). Moreover, no progression in MMSE scores was observed for any of the PD groups, indicating that these did not develop cognitive decline along the span of the study. Descriptive statistics of clinical parameters for all the groups are presented in Table 1. The patient visits per year of follow-up were: 100% for year 1 (74 participants), 82.3% for year 2 (61 participants), 77.0% (57 participants) for year 3, 63.5% (47 participants) for year 4 and 51.4% (38 participants) for year 5. An overview of data availability in the MODEP cohort is presented in the Supplementary Table A1.

### Differences Between Early Stages of PD and Healthy Control Subjects

Table 2A presents the results of the comparison between Early-PD and HCs corresponding to gait features and Mark/Subtract performances assessed in all conditions: single task (Walk, Mark and Subtract) and dual-task (W-Mark and W-Subtract). Results regarding the included confounders (Supplementary Table C1) and the effects of the time factor (Supplementary Table C2) are presented in the Appendix. In addition, Supplementary Table B1 presents means and standard deviations within groups of each gait feature and for each protocol at baseline (year 1).

**Table 2A:** Results of GEE analysis obtained for the three conditions: Walk, W-Mark, W-Subtract, and for the comparison Early-PD vs. HC.

	**Single task**				**W-mark**				**W-subtract**			
**Early-PD vs. HC**	**Group**	Group ^*^ Time	**% of annual change in HC**	**% of annual change in Early-PD**	**Group**	Group ^*^ Time	**% of annual change in HC**	**% of annual change in Early-PD**	**Group**	Group ^*^ Time	**% of annual change in HC**	**% of annual change in Early-PD**
Number of steps	0.25 (0.22 ± 0.19)	0.05 (0.05 ± 0.03)	0.36 ± 0.17%	1.03 ± 0.33%	0.10 (0.31 ± 0.19)	0.01 (0.06 ± 0.02)	0.32 ± 0.12%	0.85 ± 0.23%	0.18 (0.25 ± 0.19)	0.02 (0.07 ± 0.03)	0.14 ± 0.19%	0.91 ± 0.38%
Total duration [s]	0.00 (0.84 ± 0.25)	0.08 (0.09 ± 0.05)	0.64 ± 0.40%	1.91 ± 0.52%	0.01 (0.67 ± 0.24)	0.12 (0.09 ± 0.06)			0.00 (1.06 ± 0.24)	0.12 (0.08 ±0.05)		
Asymmetry of step time [s]	0.99 (0.00 ± 0.28)	0.18 (0.11 ± 0.08)			0.35 (0.24 ± 0.25)	0.63 (−0.04 ± 0.08)			0.78 (0.08 ± 0.29)	0.15 (−0.14 ± 0.10)		
Median stride time [s]	0.18 (−0.35 ± 0.26)	0.00 (−0.10 ± 0.03)	−0.18 ± 0.13%	−0.85 ± 0.18%	0.10 (−0.45 ± 0.27)	0.00 (−0.11 ± 0.03)	−0.07 ± 0.10%	−0.74 ± 0.15%	0.31 (−0.25 ± 0.25)	0.01 (−0.10 ± 0.04)	−0.13 ± 0.17%	−0.90 ± 0.33%
Stride time variability [s]	0.10 (0.40 ± 0.25)	0.52 (−0.05 ± 0.08)			0.38 (0.20 ± 0.23)	0.72 (−0.02 ± 0.07)			0.88 (0.04 ± 0.26)	0.69 (−0.02 ± 0.06)		
SD (Acc in VT) [m/s^2^]	0.97 (0.01 ± 0.15)	0.75 (0.01 ± 0.03)			0.59 (0.09 ± 0.17)	0.55 (0.02 ± 0.03)			0.63 (−0.07 ± 0.15)	0.92 (0.00 ± 0.03)		
SD (Acc in ML) [m/s^2^]	0.96 (0.01 ± 0.22)	0.32 (0.03 ± 0.03)			0.95 (−0.01 ± 0.24)	0.93 (0.00 ± 0.03)			0.23 (−0.25 ± 0.21)	0.33 (0.04 ± 0.04)		
SD (Acc in AP) [m/s^2^]	0.74 (−0.07 ± 0.19)	0.91 (0.00 ± 0.04)			0.25 (−0.26 ± 0.23)	0.87 (−0.01 ± 0.04)			0.28 (−0.19 ± 0.17)	0.67 (0.02 ± 0.04)		
Stride regularity (VT)	0.13 (−0.40 ± 0.26)	0.73 (−0.02 ± 0.07)			0.66 (−0.13 ± 0.30)	0.37 (−0.07 ± 0.07)			0.28 (−0.26 ± 0.24)	0.73 (0.02 ± 0.05)		
Step regularity (VT)	0.45 (−0.12 ± 0.16)	0.31 (0.14 ± 0.14)			0.64 (−0.09 ± 0.20)	0.33 (−0.05 ± 0.06)			0.96 (−0.01 ± 0.23)	0.55 (−0.06 ± 0.09)		
Stride regularity (AP)	0.00 (−0.92 ± 0.27)	0.56 (−0.04 ± 0.08)			0.05 (−0.48 ± 0.24)	0.15 (−0.11 ± 0.08)			0.03 (−0.58 ± 0.27)	0.18 (−0.07 ± 0.05)		
Step regularity (AP)	0.03 (−0.53 ± 0.24)	0.66 (0.03 ± 0.07)			0.18 (−0.29 ± 0.21)	0.24 (−0.07 ± 0.06)			0.09 (−0.39 ± 0.23)	0.93 (−0.01 ± 0.11)		
Harmonic ratio (VT)	0.38 (−0.21 ± 0.24)	0.02 (−0.13 ± 0.05)	1.68 ± 1.34%	−2.79 ± 1.21%	0.26 (−0.29 ± 0.25)	1.00 (0.00 ± 0.06)			0.33 (−0.25 ± 0.25)	0.43 (−0.05 ± 0.06)		
Harmonic ratio (ML)	0.27 (−0.25 ± 0.23)	0.00 (−0.15 ± 0.05)	−0.70 ± 0.91%	−5.93 ± 1.38%	0.07 (−0.47 ± 0.26)	0.70 (−0.03 ± 0.07)			0.02 (−0.61 ± 0.25)	0.77 (0.02 ± 0.05)		
Harmonic ratio (AP)	0.04 (−0.48 ± 0.23)	0.07 (−0.12 ± 0.06)	−1.19 ± 1.27%	−4.27 ± 1.58%	0.07 (−0.49 ± 0.27)	0.94 (0.00 ± 0.06)			0.03 (−0.60 ± 0.27)	0.98 (0.00 ± 0.07)		
Index of harmonicity (VT)	0.00 (0.63 ± 0.22)	0.04 (−0.15 ± 0.07)	0.74 ± 0.50%	−0.95 ± 0.61%	0.27 (0.33 ± 0.30)	0.16 (−0.10 ± 0.07)			0.11 (0.49 ± 0.30)	0.13 (−0.10 ± 0.07)		
Index of harmonicity (ML)	0.33 (0.26 ± 0.27)	0.25 (0.07 ± 0.06)			0.05 (0.50 ± 0.25)	0.40 (0.04 ± 0.05)			0.04 (0.56 ± 0.27)	0.86 (0.01 ± 0.05)		
Index of harmonicity (AP)	0.47 (−0.19 ± 0.26)	0.21 (0.09 ± 0.07)			0.16 (−0.34 ± 0.24)	0.86 (0.01 ± 0.07)			0.53 (−0.16 ± 0.25)	0.99 (0.00 ± 0.07)		
Normalized peak power (VT)	0.40 (0.21 ± 0.25)	0.16 (−0.11 ± 0.08)			0.47 (0.21 ± 0.29)	0.12 (−0.09 ± 0.06)			0.87 (−0.04 ± 0.23)	0.72 (−0.02 ± 0.05)		
Normalized peak power (ML)	0.11 (−0.43 ± 0.27)	0.75 (−0.02 ± 0.05)			0.36 (−0.24 ± 0.26)	0.51 (0.04 ± 0.06)			0.14 (−0.38 ± 0.26)	0.93 (0.00 ± 0.05)		
Normalized peak power (AP)	0.02 (−0.59 ± 0.26)	0.22 (−0.07 ± 0.06)			0.11 (−0.42 ± 0.26)	0.11 (−0.11 ± 0.07)			0.01 (−0.71 ± 0.28)	0.22 (−0.07 ± 0.05)		
Width of peak power (VT)	0.00 (0.91 ± 0.19)	0.83 (−0.02 ± 0.09)			0.05 (0.45 ± 0.23)	0.87 (0.01 ± 0.08)			0.02 (0.59 ± 0.24)	0.38 (−0.07 ± 0.08)		
Width of peak power (ML)	0.04 (0.39 ± 0.19)	0.61 (−0.05 ± 0.10)			0.76 (−0.09 ± 0.30)	0.70 (0.03 ± 0.08)			0.76 (−0.09 ± 0.30)	0.91 (−0.01 ± 0.09)		
Width of peak power (AP)	0.02 (0.49 ± 0.22)	0.31 (−0.09 ± 0.08)			0.07 (0.44 ± 0.24)	0.95 (0.00 ± 0.08)			0.03 (0.77 ±0.35)	0.11 (−0.19 ± 0.12)		
Cognitive and motor performance: box marking	0.02 (−0.57 ± 0.25)	0.31 (−0.04 ± 0.04)			0.85 (0.04 ± 0.23)	0.85 (−0.01 ± 0.05)						
Cognitive performance: subtracting −7	0.08 (−0.49 ± 0.28)	0.02 (−0.10 ± 0.04)	−0.06 ± 0.36%	−1.92 ± 0.77%					0.40 (−0.20 ± 0.23)	0.24 (−0.14 ± 0.12)		

The table presents p-values, followed by β-values ± standard errors for effects of Time, Group, and the interaction Time

* Group. All β-values are based on transformed and normalized data (log transformation and z-score normalization). In addition, the percentage of annual change relative to the first year is presented for each group for those features that were affected by the interaction Time*Group. The percentage of annual change is based on non-transformed and non-normalized data. SD, standard deviation; VT, vertical acceleration; ML, medio-lateral acceleration; AP, anterior-posterior acceleration; Walk, single task walking in circles; W-Mark, walking in circles while marking-crosses in the paper sheet; W-Subtract, walking in circles while subtracting serial 7 digits.

*
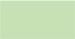
Result with trend p < 0.1 (expected direction)*.

*
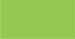
Significant result with p < 0.05 (expected direction)*.

At baseline (Supplementary Table B1), the Early-PD walked slower (longer duration) than the HC, not only in the single task Walk, but also in both dual-tasking protocols, W-Mark and W-Subtract. W-Subtract presented the highest β-value (1.06 ± 0.24) among the three protocols. Only under single task condition Walk, a trend (*p* = 0.08) toward a significant (group x time) interaction on duration was found with more increase in Early-PD than HC (1.91% vs 0.64%). The number of steps and the median stride time changed faster in the Early-PD than in the HC, with similar ratios of progression among the three protocols (i.e., β-values for the interaction group x time ranging from 0.05 to 0.07 in number of steps and from −0.11 to −0.10 in median stride time). Moreover, annual percentages of change in the Early-PD group were comparable among the three protocols, with the highest annual change in number of steps for the single task Walk (1.03%), and in stride time for the W-Subtract (−0.90%).

Although some of the other gait features obtained in dual-tasking protocols showed differences or trends toward difference between Early-PD and HC at baseline (Supplementary Table B1, stride regularity AP, harmonic ratios, index of harmonicity ML, normalized peak power AP, width of peak power), none of these showed a significant difference in progression between Early-PD and HC.

Performances of Mark and Subtract tasks only reached significance in single tasking. For instance, the Early-PD (Table 2A) presented worse in marking crosses at baseline and worsened more rapidly than HC in the single task Subtract (−1.92% annual change, β-value: −0.10 ± 0.04).

**Table 3A** presents group differences in DTI between Early-PD and HC under both dual-tasking conditions. Results regarding the effects on the time factor (Supplementary Table C3) are presented in the Appendix. For trial duration, a negative β-value, indicating a smaller increase in trial duration due to the dual task at baseline in the Early-PD than HC was observed for W-Mark (−0.35 ± 0.16), but a positive β-value was found for W-Subtract (0.60 ± 0.21). Harmonic ratio VT and index of harmonicity VT presented lower DTI under both dual-task conditions in the Early-PD relative to HC at baseline; the same was observed for harmonic ratios ML and AP in the W-Subtract condition. Moreover, when calculating DTI independently from task prioritization (S-DTI), lower or a trend toward lower S-DTIs (i.e., higher costs) for the Early-PD than for the HC in index of harmonicity VT and harmonic ratios (VT, ML and AP) were found at baseline in the W-Mark condition. Notice that the sign of DTI% on total duration (Early-PD) was inverted to calculate S-DTI.

Surprisingly, only few group x time interactions on DTI and S-DTI were found, and in fact each of them were opposite to the expected direction, and opposite to annual changes of non-transformed data. Specifically, harmonic ratio in ML presented positive β-values for the interaction of group and time of 0.23 ± 0.10 (W-Mark) and 0.21 ± 0.08 (W-Subtract). These had an opposite direction to annual changes obtained for the Early-PD from non-transformed data: −34.16% (W-Mark) and −30.46% (W-Subtract), while no significant annual changes were observed for the HC group. Similar β-values were obtained for the interaction of group and time in S-DTI of the same feature in W-Mark (0.21 ± 0.10), again, opposite to the percentages of annual change obtained from non-transformed data (−18.18% in Early-PD and no significant annual change in HC).

### Differences Between Middle Stages of PD and Healthy Control Subjects

As for the single task, most of the gait features obtained from dual-tasking protocols did present group differences at baseline between the Mid-PD and the HC (Table 2B and Supplementary Table B1). Specifically, the Mid-PD, compared to the reference group, used a larger number of steps, while trial duration was longer, and presented higher asymmetry in step time, lower SD in AP, lower step regularity in AP, lower harmonic ratios, higher index of harmonicity and higher width of peak power (this feature only in Walk and W-Subtract protocols). Trial duration, asymmetry in step time and AP harmonic ratio yielded the highest beta values, especially for W-Mark.

**Table 2B:** Results of GEE analysis obtained for the three conditions: Walk, W-Mark, W-Subtract, and for the comparison Mid-PD vs. HC.

	**Single task**				**W-mark**				**W-subtract**			
**Mid-PD vs. HC**	**Group**	Group * Time	**% of annual change in HC**	**% of annual change in Early-PD**	**Group**	Group * Time	**% of annual change in HC**	**% of annual change in Early-PD**	**Group**	Group * Time	**% of annual change in HC**	**% of annual change in Early-PD**
Number of steps	0.00 (0.54 ± 0.14)	0.47 (−0.02 ± 0.03)			0.00 (0.46 ± 0.13)	0.81 (−0.01 ± 0.03)			0.00 (0.31 ± 0.10)	0.69 (−0.01 ± 0.03)		
Total duration [s]	0.00 (0.89 ± 0.22)	0.49 (0.06 ± 0.09)			0.00 (1.00 ± 0.25)	1.00 (0.00 ± 0.05)			0.00 (0.98 ± 0.23)	0.41 (0.03 ± 0.04)		
Asymmetry of step time [s]	0.02 (0.50 ± 0.21)	0.34 (0.07 ± 0.08)			0.00 (0.79 ± 0.23)	0.63 (−0.03 ± 0.07)			0.00 (0.70 ± 0.23)	0.17 (−0.12 ± 0.09)		
Median stride time [s]	0.65 (−0.11 ± 0.24)	0.28 (−0.06 ± 0.05)			0.01 (−0.60 ± 0.24)	0.99 (0.00 ± 0.06)			0.54 (−0.18 ± 0.29)	0.39 (0.06 ± 0.07 0029		
Stride time variability [s]	0.72 (−0.08 ± 0.22)	0.01 (0.20 ± 0.07)	−1.34 ± 1.97%	6.09 ± 1.90%	0.15 (−0.36 ± 0.25)	0.02 (0.19 ± 0.08)	−1.68 ± 2.30%	4.69 ± 2.74%	0.20 (−0.31 ± 0.24)	0.00 (0.17 ± 0.06)	−2.71 ± 2.04%	4.33 ± 2.20%
SD (Acc in VT) [m/s^2^]	0.01 (−0.48 ± 0.20)	0.41 (0.02 ± 0.03)			0.68 (−0.06 ± 0.15)	0.74 (−0.01 ± 0.02)			0.19 (−0.23 ± 0.17)	0.72 (−0.01 ± 0.03)		
SD (Acc in ML) [m/s^2^]	0.21 (−0.25 ± 0.20)	0.90 (−0.01 ± 0.04)			0.90 (0.03 ± 0.23)	0.00 (−0.11 ± 0.03)	1.81 ± 0.87%	−2.22 ± 0.94%	0.36 (−0.18 ± 0.20)	0.03 (−0.08 ± 0.04)	1.28 ± 1.31%	−2.07 ± 1.17%
SD (Acc in AP) [m/s^2^]	0.01 (−0.53 ± 0.20)	0.30 (0.04 ± 0.04)			0.01 (−0.41 ± 0.16)	0.60 (−0.02 ± 0.04)			0.01 (−0.45 ± 0.17)	0.73 (−0.01 ± 0.04)		
Stride regularity (VT)	0.01 (0.51 ± 0.21)	0.05 (−0.15 ± 0.07)	−1.23 ± 0.43%z	−2.21 ± 0.71%	0.03 (0.45 ± 0.20)	0.24 (−0.08 ± 0.07)			0.04 (0.34 ± 0.17)	0.20 (−0.06 ± 0.05)		
Step regularity (VT)	0.06 (−0.30 ± 0.16)	0.27 (0.16 ± 0.14)			0.55 (−0.12 ± 0.20)	0.74 (0.03 ± 0.09)			0.74 (−0.05 ± 0.16)	0.48 (0.05 ± 0.08)		
Stride regularity (AP)	0.08 (−0.39 ± 0.23)	0.01 (−0.16 ± 0.06)	−1.78 ± 0.67%	−4.34 ± 0.82%	0.20 (−0.37 ± 0.29)	0.04 (−0.14 ± 0.07)	−0.57 ± 0.47%	−2.90 ± 0.83%	0.17 (−0.36 ± 0.27)	0.00 (−0.14 ± 0.05)	−0.64 ± 0.70%	−4.02 ± 0.87%
Step regularity (AP)	0.07 (−0.36 ± 0.20)	0.16 (0.17 ± 0.12)			0.04 (−0.42 ± 0.20)	0.39 (0.06 ± 0.07)			0.06 (−0.39 ± 0.21)	0.28 (0.13 ± 0.12)		
Harmonic ratio (VT)	0.01 (−0.51 ± 0.19)	0.07 (−0.10 ± 0.06)	1.80 ± 1.35%	−1.55 ± 1.50%	0.00 (−0.74 ± 0.19)	0.61 (−0.03 ± 0.07)			0.00 (−0.75 ± 0.21)	0.48 (−0.05 ± 0.07)		
Harmonic ratio (ML)	0.03 (−0.53 ± 0.24)	0.28 (0.06 ± 0.06)			0.18 (−0.37 ± 0.28)	0.84 (−0.01 ± 0.07)			0.01 (−0.67 ± 0.25)	0.62 (0.02 ± 0.05)		
Harmonic ratio (AP)	0.00 (−0.86 ± 0.21)	0.54 (0.04 ± 0.07)			0.00 (−1.07 ± 0.21)	0.13 (0.10 ± 0.07)			0.00 (−1.08 ± 0.21)	0.47 (0.04 ± 0.06)		
Index of harmonicity (VT)	0.00 (0.89 ± 0.25)	0.23 (−0.08 ± 0.07)			0.01 (0.68 ± 0.26)	0.13 (−0.10 ± 0.06)			0.02 (0.43 ± 0.19)	0.72 (−0.02 ± 0.04)		
Index of harmonicity (ML)	0.57 (0.15 ± 0.27)	0.48 (−0.05 ± 0.08)			0.35 (0.24 ± 0.25)	0.51 (−0.05 ± 0.08)			0.12 (0.44 ± 0.28)	0.68 (−0.03 ± 0.07)		
Index of harmonicity (AP)	0.03 (0.47 ± 0.22)	0.77 (0.01 ± 0.04)			0.79 (0.06 ± 0.23)	0.92 (−0.01 ± 0.07)			0.02 (0.56 ± 0.24)	0.33 (0.06 ± 0.06)		
Normalized peak power (VT)	0.03 (0.43 ± 0.20)	0.10 (−0.11 ± 0.07)	−0.98 ± 1.02%	−2.02 ± 0.84%	0.07 (0.35 ± 0.19)	0.09 (−0.10 ± 0.06)	−1.50 ± 0.77%	−2.27 ± 1.00%	0.37 (0.15 ± 0.16)	0.07 (−0.07 ± 0.04)	−1.88 ± 0.77%	−2.70 ± 0.61%
Normalized peak power (ML)	0.01 (−0.57 ± 0.23)	0.63 (0.04 ± 0.07)			0.28 (−0.25 ± 0.23)	0.97 (0.00 ± 0.09)			0.17 (−0.36 ± 0.26)	0.71 (0.03 ± 0.07)		
Normalized peak power (AP)	0.42 (−0.18 ± 0.22)	0.59 (−0.03 ± 0.06)			0.79 (−0.06 ± 0.24)	0.30 (−0.07 ± 0.06)			0.24 (−0.27 ± 0.23)	0.75 (−0.02 ± 0.06)		
Width of peak power (VT)	0.00 (0.85 ± 0.19)	0.70 (0.03 ± 0.08)			0.99 (0.00 ± 0.28)	0.81 (−0.02 ± 0.06)			0.01 (0.63 ± 0.23)	0.51 (−0.05 ± 0.08)		
Width of peak power (ML)	0.02 (0.55 ± 0.24)	0.24 (−0.14 ± 0.12)			0.97 (−0.01 ± 0.31)	0.33 (0.12 ± 0.12)			0.82 (−0.07 ± 0.30)	0.14 (0.12 ± 0.08)		
Width of peak power (AP)	0.11 (−0.35 ± 0.22)	0.12 (0.15 ± 0.09)			0.28 (0.28 ± 0.26)	0.92 (0.01 ± 0.09)			0.07 (0.49 ± 0.27)	0.57 (−0.05 ± 0.09)		
Cognitive and motor performance: box marking	0.00 (−1.05 ± 0.23)	0.41 (0.04 ± 0.04)			0.09 (0.37 ± 0.21)	0.08 (−0.08 ± 0.05)	84.63 ± 37.73%	9.65 ± 13.39%				
Cognitive performance: subtracting −7	0.02 (−0.41 ± 0.18)	0.71 (0.04 ± 0.10)							0.16 (−0.26 ±0.19)	0.16 (−0.12 ± 0.09)		

The table presents p-values, followed by β-values ± standard errors for effects of Time, Group and the interaction Time*Group. All β-values are based on transformed and normalized data (log transformation and z-score normalization). In addition, the percentage of annual change relative to the first year is presented for each group for those features that were affected by the interaction Time*Group. The percentage of annual change is based on non-transformed and non-normalized data. SD, standard deviation; VT, vertical acceleration; ML, medio-lateral acceleration; AP, anterior-posterior acceleration; Walk, single task walking in circles; W-Mark, walking in circles while marking-crosses in the paper sheet; W-Subtract, walking in circles while subtracting serial 7 digits.

*
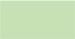
Result with trend p < 0.1 (expected direction)*.

*
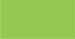
Significant result with p < 0.05 (expected direction)*.

*
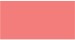
Result with trend p < 0.1 (unexpected direction)*.

*
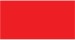
Significant result with p < 0.05 (unexpected direction)*.

However, only three gait features, common among both dual-tasking protocols, reflected consistent differences in progression between groups (stride time variability, stride regularity in AP and SD in ML). β-values for the interaction group x time on stride time variability were similar (ranging between 0.17 and 0.20) among the three protocols. Annual percentages of change were only significant in the Mid-PD, with similar values among both dual-tasking protocols: 4.69% (W-Mark) and 4.33% (W-Subtract), and slightly higher values for the single task: 6.09%. Also AP stride regularity decreased faster in the Mid-PD than in the HC under all three conditions (with β-values of −0.14 in both dual-tasking protocols and −0.16 in single task); and with significant annual changes only in the Mid-PD group: −4.34% (Walk), −2.90 (W-Mark) and −4.02 (W-Subtract). Differences between groups in progression of VT stride regularity only approached significance in the single-task condition. Likewise, the group x time interaction for the VT harmonic ratio only approached significance in the single task condition, with a faster decrease in the Mid-PD group compared to HC. However, no significant annual changes were observed in non-transformed data for any of the groups.

On the other hand, SD of ML acceleration showed a faster decrease in the Mid-PD compared to HC only in dual-task conditions, with similar β-values (−0.11 ± 0.03 in W-Mark and −0.08 ± 0.04 in W-Subtract), similar percentages of annual change in the Mid-PD group (−2.22% and −2.07%, respectively) and no significant annual changes in the reference group. This feature did not present progression differences between groups when measured in the single-task condition, although group differences were found at baseline for the SD of VT and AP acceleration.

For the VT normalized peak power, a trend toward progression differences between groups was observed in both dual-tasking protocols, with β-values of −0.10 (W-Mark) and −0.07 (W-Subtract) and similar percentages of annual change in the Mid-PD group: −2.27% (W-Mark) and −2.70% (W-Subtract), and in HC: −1.50 (W-Mark) and −1.88 (W-Subtract). It must be noted that this feature showed, opposite to expectations, higher values at baseline in the Mid-PD group than in the HC in Walk and W-Mark conditions. Likewise, the analysis indicated an unexpectedly higher VT stride regularity at baseline in the Mid-PD than in the HC for all three conditions. However, when removing corrections for confounders, lower β-values were observed for the Mid-PD in stride regularity VT (W-Mark, *p* = 0.07 and W-Subtract, *p* = 0.01) and normalized peak power VT (W-Subtract, *p* = 0.01), which suggests an over-compensation in the original results, mainly caused by gait speed.

At baseline (Supplementary Table B1), the Mid-PD group performed both Mark and Subtract single tasks worse than HC. However, when dual-tasking, the Mid-PD tended to mark crosses (*p* = 0.09) better than the control group (Table 2B). In the Mid-PD, performance in marking crosses under dual-tasking decreased faster than in HC (β-value: −0.08 ± 0.05, with an 84.63% of annual change in the HC and non-significant annual change in Early-PD).

At baseline, lower DTIs (higher dual-tasking costs) were found in the Mid-PD than in the HC (**Table 3B**) for VT normalized peak power and VT harmonic ratio (in both dual-task protocols), marking crosses, SD of ML (W-Mark) and stride VT regularity (W-Subtract). Although positive β-values for group effects (0.52 ± 0.21), indicating higher costs for the Mid-PD at baseline, were obtained for the DTI of SD (ML), negative values (−0.11 ± 0.05) were found in the group x time interaction under W-Mark condition, indicating a decreasing difference over time, with significant annual changes only in the Mid-PD of 7.12%. For S-DTI no baseline or group x time interactions were found for the W-Subtract condition. For the W-Mark condition, lower values (higher costs) at baseline in the Mid-PD were observed for SD of ML, stride regularity in VT and AP, harmonic ratio VT and normalized peak power VT. Notice that the sign of DTI% on SD of ML (Mid-PD) was inverted to calculate S-DTI.

### Potential Markers of Progression in Parkinson‘s Disease

**Figure 2:**
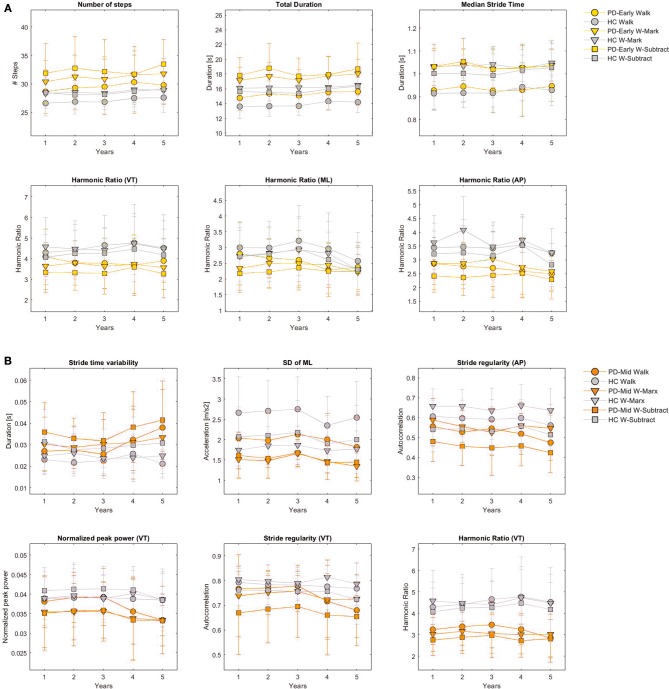
Changes on gait features selected as progression markers over follow-up. Averaged biannual values and standard errors over the 5 years of follow-up for the features identified as progression markers of early stages PD **(A)** and middle stages PD **(B)** are presented for the three protocols (Walk, W-Subtract, W-Mark), for the HC and the Early-PD groups **(A)** and for the HC and the Mid-PD groups **(B)**. This figure is based on non-transformed and non-normalized (z-score) data. SD, standard deviation; VT, vertical acceleration; ML, medio-lateral acceleration; AP, anterior-posterior acceleration; Walk, single task walking in circles; W-Mark, walking in circles while marking-crosses in the paper sheet; W-Subtract, walking in circles while subtracting serial 7 digits.

Based on the criteria defined in the methods section, the number of steps was identified as a potential progression marker of early PD in all three protocols. In single task walking, as already reported (26), also the total duration and the harmonic ratios in the three directions (VT, ML, and AP) fulfilled the criteria. For W-Mark, the median stride time can also be considered as a progression marker in early PD. Figure 2A presents non-transformed averaged biannual values of the 5 years of follow-up for features identified as progression markers of early stages PD. This is presented for the three protocols and for the HC and the Early-PD groups.

For the Mid-PD group, potential progression markers common among the three protocols were: stride time variability and stride regularity AP. In single task walking, also VT stride regularity and VT harmonic ratio fulfilled the criteria, whereas SD of ML and normalized peak power VT could be considered as progression markers of mid-stage PD when assessed under W-Mark or W-Subtract dual-tasking conditions. Figure 2B presents non-transformed averaged biannual values of the 5 years of follow-up for features identified as progression markers of middle stages PD. This is presented for the three protocols and for the HC and the Mid-PD groups.

None of the DTI, nor S-DTI on gait features fulfilled the criteria for progression marker of any stage of PD.

## Discussion

This study investigated the added value of quantitative assessment of circular gait in dual-task walking, as compared to single-task walking, in assessment of progression of early and middle stages of PD. We hypothesized that more gait features would be identified as progression markers of the disease under dual-task conditions. However, the results did not confirm our hypothesis; fewer gait features from dual-task assessments were identified as markers of progression in PD. Moreover, against our expectations, while some baseline group differences in dual-task interference between PD groups and HC were found, we did not find clear evidence of worsening of dual-task interference in patients with PD. On the other hand, the results support the validity and robustness of some of the gait features already identified as progression markers of the disease in a single-task circular walking condition (26).

### Differences Between Gait Features From Single and Dual-Task Conditions

Fewer gait features extracted from dual-tasking protocols than from single-task walking fulfilled the criteria for progression markers of PD. Particularly, in early stages of PD only one feature, number of steps, was commonly identified as a progression marker in all three protocols. Lower consistency of gait features from dual-tasking as progression markers of early PD could be due to many individual factors and available resources that can influence the magnitude and pattern of dual-task interference on gait performance and can introduce inter- and intra-subject variability in the data (22). These factors could include the patient's attention to the execution of the concurrent tasks (13), understanding of instructions, motivation, confidence in physical capabilities and arousal (8, 10). On the other hand, these findings underline the robustness of step/stride cycles as a progression marker compared to other gait features. Generally, step length is decreased and cadence is increased in PD, which is considered the central motor impairment in hypokinetic gait, possibly related to bradykinesia (47–49).

Although more gait features obtained from W-Subtract presented group differences between PD and HC than features from W-Mark, the median stride time was only significantly different between the Mid-PD and the HC under W-Mark condition, which contributed to fulfilling the criteria of progression marker in the Early-PD for this feature. Moreover, under W-Mark, stride time approached significance for the comparison between Early-PD and HC. The visuomotor task (marking crosses) implies deprivation of visual information on the walking path and on the individuals' own feet (21) when performed simultaneously with a walking task. Thus, W-Mark might have led to shorter strides as a consequence of such physical interference in addition to the limited cognitive resources available for the walking task (attention, visuospatial and executive functions) (1, 22).

For middle stages of PD, a more comparable number of features were identified as progression markers among single and dual-tasking protocols. This reinforces the validity and robustness of two of the gait features already identified as progression markers of the disease in single-task circular walking: stride time variability and stride regularity AP; features reflecting progressive decrement of regularity and consistency of the gait pattern (26). These are common impairments in PD, present in walking while turning (4, 50), reflecting a progressive loss of automaticity with disease progression (11).

The identification of more progression markers for middle stages than for early stages of PD when assessing dual-tasking conditions could be caused by the fact that gait impairments are less pronounced in early stages of the disease (51). In addition, compensation strategies may have been differently addressed between visits among individuals at early-stage PD, thereby causing high intra-subject variability in the Early-PD and a lack of consistent differences between Early-PD and HC. Moreover, compensation strategies might be more often present in early than in middle phases of basal ganglia degeneration (27). Although we expected to reveal progressive deficits unnoticed under single-task walking by assessing dual-task protocols, compensatory mechanisms might still have been present under dual-tasking protocols, masking pathological gait impairments (27).

Under dual-tasking, features related to ML movement (SD of ML, index of harmonicity in ML and normalized peak power VT) showed significance, while for single walking they did not. Thus, the sensitivity of dual-task performance to impairments related to PD was mainly reflected in features extracted from ML accelerations. For instance, the results indicated that already in early stages of PD patients walk with smoother lateral movement (higher index of harmonicity in ML) and a more acyclic and asymmetrical stride-to-stride pattern than the HC under dual-task conditions, followed by a progressive reduction of lateral gait intensity (SD of ML acceleration) at middle stages. A smoother and less intense gait pattern may reflect a more cautious gait performance (41).

Although at baseline and under W-Mark condition (Table 2B) the Mid-PD group marked more boxes than the HC (only approaching significance, and potentially due to task prioritization), the percentages of annual changes showed an improvement on this task only for the HC group. Moreover, the interaction term reflected a progressive and relative improvement on this task for the controls with respect the Mid-PD. These could have potentially resulted as a consequence of learning in the HC, which were not observed in the Mid-PD.

### Dual-Task Interference

Both PD groups presented at baseline higher dual task costs on gait performance of W-Mark and W-Subtract than HC (DTIs of total duration, harmonic ratios and index of harmonicity for the comparison Early-PD vs. HC, and SD of ML, harmonic ratio and normalized peak power for the comparison Mid-PD vs. HC). However, only under W-Mark and independently of prioritization aspects, both PD groups still presented higher costs than HC (as reflected by negative β-values of S-DTI). This is in agreement with a higher cost on marking crosses found in the Mid-PD with respect the HC. Mutual interferences suggest that the cognitive, motor and executive functions required to mark crosses while walking might demand more attentional resources than the total capacity available in the Mid-PD, especially in comparison to single performance of either task (8). Consequently, both tasks deteriorated, although not to the same degree. This can be explained by the “capacity sharing” model of dual-task interference (52), which states that tasks can be performed in parallel despite limited available resources to perform each task. However, it must be noted that the Early-PD presented higher positive DTI on duration of trial under W-Mark than the HC. This suggests that the Early-PD might have already required a longer time to perform the trial under single-task walking than the HC, and thereby, the difference with respect dual-tasking was not as pronounced in Early-PD as for the HC.

For the comparison of both PD groups with respect HC under W-Subtract, only interferences in gait performance (DTI on total duration, harmonic ratios and index of harmonicity in the comparison Early-PD vs. HC and DTI on SD of ML, stride regularity, harmonic ratio and normalized peak power in VT for Mid-PD vs. HC), and not in performance of the Subtract task, were observed, possibly suggesting limited cognitive resources in PD, but with a latent prioritization of the cognitive task to the detriment of the walking task (posture second strategy) (8, 21, 24). This could be explained by the fact that under challenging dual-task conditions, individuals with limited cognitive resources (deprived flexibility and memory) focus their attention on the most challenging task (24). Particularly, considering that counting backwards essentially depends on the working memory and is related to executive functions (5), individuals with cognitive impairments often present higher dual-task interferences on the walking task and try to perform better at the subtraction task than individuals without cognitive decline (6, 7, 24, 33, 53). This could have been the case in both PD groups, who clearly showed limited cognitive and executive functions at baseline, as indicated by a poorer performance of the single Mark and Subtract tasks than HC.

We observed in DTI (harmonic ratio ML and AP under W-Subtract and harmonic ratio ML under W-Mark) and S-DTI (harmonic ratio ML under W-Mark) an inconsistent opposite direction between the β-values obtained for the interaction group x time and the percentage of annual changes obtained separately for each of the groups when comparing Early-PD vs. HC. The same inconsistent results were observed for DTI (SD of ML under W-Mark) when comparing Mid-PD vs. HC. Focusing uniquely on the percentages of annual change, it seems that PD patients presented a significant increase of DTI reflected in harmonic ratio ML for Early-PD and in SD of ML for the Mid-PD. This leads to the conclusion that based on these data we cannot consistently demonstrate a progressive dual-task-interference change in patients with PD. Lack of consistent differences in progression between groups on DTI and on S-DTI could be due to the fact that walking in circles is already a difficult/challenging task for patients with PD (2). Moreover, there might be different prioritization strategies among patients influenced by the walking path, i.e., by the circular trajectory (24). Thus, against our expectations, dual-tasking assessment leads to non-uniform effects among gait features (22) and might not add significant information regarding the progression of the disease under the conditions of this study. In addition, variance in nature, severity and progression rate of motor and cognitive impairments (10, 19) as well as transient variations in effective capacity due to factors like motivation, emotional disequilibrium (e.g., depression), fatigue, sleepiness and arousal (8, 10, 54) may have contributed to variability in dual-task costs.

The DTI on index of harmonicity VT was lower in early-stage PD than in HCs under both dual-tasking conditions, which indicates that a dual-task condition might have induced a more erratic movement compared to the single task. Although patients at early-stage PD presented a higher index of harmonicity VT at baseline than HC (see Table 3A, a positive β-value for group effect, reflecting a smoother pattern), these patients performed the gait task with higher frequency components under a dual-tasking condition.

**Table 3A:** Results of GEE analysis obtained for dual-task-interference (DTI) and summed dual-task-interference (S-DTI) for conditions: W-Mark and W-Subtract for the comparison Early-PD vs. HC.

	**Early-PD vs. HC Dual-Task Interference**	**W-MARK**			**W-SUBTRACT**		
		**Confounders**	**Group**	Group* time	**Confounders**	**Group**	Group* time
DTI	Number of steps	a	0.51 (0.10 ± 0.15)	0.66 (−0.03 ± 0.06)	a/c	0.23 (0.23 ± 0.19)	0.37 (0.06 ± 0.06)
	Total duration	b/c	0.03 (−0.35 ± 0.16)	0.42 (−0.09 ± 0.11)	b	0.01 (0.60 ± 0.21)	0.67 (−0.04 ± 0.10)
	Median stride time	a	0.76 (0.04 ± 0.12)	0.11 (−0.12 ± 0.07)	a	0.50 (−0.10 ± 0.14)	0.80 (−0.02 ± 0.08)
	Harmonic ratio (VT)	a	0.04 (−0.54 ± 0.25)	0.38 (0.09 ± 0.10)	0	0.03 (−0.53 ± 0.25)	0.44 (0.07 ± 0.10)
	Harmonic ratio (ML)	0	0.15 (−0.37 ± 0.26)	0.02 (0.23 ± 0.10)	b	0.02 (−0.56 ± 0.24)	0.01 (0.21 ± 0.08)
	Harmonic ratio (AP)	0	0.11 (−0.33 ± 0.21)	0.22 (0.15 ± 0.12)	b	0.04 (−0.59 ± 0.29)	0.08 (0.15 ± 0.08)
	Index of harmonicity (VT)	a/c	0.01 (−0.58 ± 0.23)	0.29 (0.09 ± 0.08)	c	0.00 (−0.70 ± 0.17)	0.75 (−0.03 ± 0.11)
	Cognitive / Cognitive and motor performance	0	0.15 (−0.33 ± 0.23)	0.13 (0.09 ± 0.06)	b	0.86 (−0.04 ± 0.23)	0.86 (0.02 ± 0.10)
S-DTI	Number of steps	a	0.16 (−0.33 ± 0.24)	0.22 (0.08 ± 0.06)	b	0.50 (0.18 ± 0.27)	0.59 (−0.07 ± 0.13)
	Total duration	c	0.89 (0.04 ± 0.28)	0.40 (0.04 ± 0.05)	b	0.40 (−0.23 ± 0.27)	0.92 (0.01 ± 0.11)
	Median stride time	a	0.26 (−0.27 ± 0.24)	0.22 (0.07 ± 0.06)	b	0.58 (0.14 ± 0.25)	0.82 (−0.03 ± 0.11)
	Harmonic ratio (VT)	0	0.03 (−0.62 ± 0.28)	0.25 (0.12 ± 0.10)	0	0.45 (−0.19 ± 0.26)	0.78 (0.03 ± 0.10)
	Harmonic ratio (ML)	0	0.06 (−0.49 ± 0.26)	0.05 (0.21 ± 0.10)	0	0.48 (−0.17 ± 0.24)	0.37 (0.09 ± 0.10)
	Harmonic ratio (AP)	0	0.03 (−0.45 ± 0.20)	0.19 (0.15 ± 0.12)	0	0.35 (−0.24 ± 0.26)	0.33 (0.08 ± 0.08)
	Index of harmonicity (VT)	b/c	0.05 (−0.51 ± 0.26)	0.18 (0.09 ± 0.07)	a/b	0.90 (0.03 ± 0.26)	0.80 (−0.03 ± 0.11)

The table presents p-values followed by β-values ± standard errors for effects of Time, Group and the interaction Time*Group. All β-values are based on transformed and normalized data (log transformation and z-score normalization). In addition, the percentage of annual change relative to the first year is presented for each group for those features that were affected by the interaction Time*Group. The percentage of annual change is based on non-transformed and non-normalized data. Significant confounders which were accounted for in the analysis are indicated as follows: “a” for DTI or S-DTI on gait speed, “b” for age effect and “c” for ON/OFF medication state effect. SD, standard deviation; VT, vertical acceleration; ML,medio-lateral acceleration; AP, anterior-posterior acceleration; W-Mark, walking in circles while marking-crosses in the paper sheet; W-Subtract, walking in circles while subtracting serial 7 digits.

*
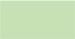
Result with trend p < 0.1 (expected direction)*.

*
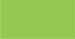
Significant result with p < 0.05 (expected direction)*.

*
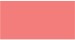
Result with trend p < 0.1 (unexpected direction)*.

*
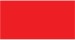
Significant result with p < 0.05 (unexpected direction)*.

Similarly, DTI on SD of ML was higher for the Mid-PD than for HC, reflecting a more intense gait pattern under dual-tasking than in single-tasking. However, Mid-PD patients progressively reduced the SD of ML acceleration (relative to HC) of their gait performance under a dual-tasking condition (see Table 3B, a negative β-value for the interaction group x time). All of these findings suggest that although patients with PD perform an overall smoother and less intense gait pattern than HC, when comparing dual-task with single-task, the effect of dual-task is more pronounced in the PD group and induces a more erratic and intense gait pattern. This would potentially indicate the adoption of a “posture second strategy” in dual-task performance (21). However, when comparing the performance of dual-tasking relative to single tasking (DTI on SD of ML) for the comparison Mid-PD vs. HC, we observed a higher frequency and more intense gait pattern in PD, which can be due to the “constraint” or “limited degrees of freedom available in the system” that challenge the control of the dynamic balance and impose the adoption of a different gait strategy (41). On the other hand, high or low values of the same features can reflect “good” or “bad” quality of gait depending on the conditions of the assessment. Thus, findings based on the proposed gait features must be carefully interpreted, considering all confounding effects and other factors and conditions.

**Table 3B:** Results of GEE analysis obtained for dual-task-interference (DTI) and summed dual-task-interference (S-DTI) for conditions: W-Mark and W-Subtract for the comparison Early-PD vs. HC.

	**Mid-PD vs. HC Dual-Task Interference**	**W-MARK**			**W-SUBTRACT**		
		**Confounders**	**Group**	Group ^*^ Time	**Confounders**	**Group**	Group ^*^ Time
DTI	Stride time variability	0	1.00 (0.00 ± 0.19)	0.71 (−0.03 ± 0.09)	a	0.82 (−0.06 ± 0.27)	0.36 (−0.09 ± 0.09)
	SD (ML)	a/b	0.01 (0.52 ± 0.21)	0.04 (−0.11 ± 0.05)	a	0.09 (0.45 ± 0.26)	0.15 (−0.13 ±- 0.09)
	Stride regularity (VT)	0	0.11 (−0.56 ± 0.35)	0.33 (0.10 ± 0.11)	a	0.01 (−0.49 ± 0.20)	0.10 (0.10 ± 0.06)
	Stride regularity (AP)	0	0.19 (−0.25 ± 0.19)	0.69 (−0.04 ± 0.11)	0	0.33 (−0.23 ± 0.24)	0.32 (−0.06 ± 0.06)
	Harmonic ratio (VT)	0	0.02 (−0.54 ± 0.24)	0.51 (0.06 ± 0.10)	a	0.06 (−0.39 ± 0.21)	0.51 (0.07 ± 0.11)
	Normalized peak power (VT)	0	0.04 (−0.65 ± 0.31)	0.75 (0.03 ± 0.09)	a	0.00 (−0.68 ±0.20)	0.44 (0.05 ± 0.06)
	Cognitive / Cognitive and motor performance	c	0.00 (−0.78 ± 0.22)	0.99 (0.00 ± 0.10)	0	0.28 (0.10 ± 0.10)	0.23 (−0.12 ± 0.10)
S-DTI	Stride time variability	0	0.16 (−0.24 – 0.17)	0.26 (0.09 ± 0.08)	a	0.71 (0.08 ± 0.21)	0.30 (−0.12 ± 0.11)
	SD (ML)	a/c	0.00 (−1.15 ± 0.26)	0.20 (0.11 ± 0.09)	0	0.82 (0.03 ± 0.14)	0.25 (−0.11 ± 0.10)
	Stride regularity (VT)	a/b/c	0.00 (−1.18 ± 0.21)	0.42 (0.07 ± 0.09)	a	0.70 (−0.06 ± 0.15)	0.27 (−0.11 ± 0.10)
	Stride regularity (AP)	c	0.00 (−0.88 ± 0.30)	0.78 (0.03 ± 0.11)	0	0.97 (0.01 ± 0.14)	0.18 (−0.14 ± 0.11)
	Harmonic ratio (VT)	a	0.00 (−0.73 ± 0.19)	0.50 (0.07 ± 0.11)	0	0.63 (−0.07 ± 0.15)	0.29 (−0.11 ± 0.10)
	Normalized peak power (VT)	a/c	0.00 (−1.10 ± 0.22)	0.78 (0.02 ± 0.08)	0	0.21 (−0.19 ± 0.15)	0.21 (−0.13 ± 0.10)

The table presents p-values followed by β-values ± standard errors for effects of Time, Group, and the interaction Time

* Group. All β-values are based on transformed and normalized data (log transformation and z-score normalization). In addition, the percentage of annual change relative to the first year is presented for each group for those features that were affected by the interaction Time*Group. The percentage of annual change is based on non-transformed and non-normalized data. Significant confounders which were accounted for in the analysis are indicated as follows: “a” for DTI or S-DTI on gait speed, “b” for age effect and “c” for ON/OFF medication state effect. SD, standard deviation; VT, vertical acceleration; ML, medio-lateral acceleration; AP, anterior-posterior acceleration; W-Mark, walking in circles while marking-crosses in the paper sheet; W-Subtract, walking in circles while subtracting serial 7 digits.

*
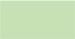
Result with trend p < 0.1 (expected direction)*.

*
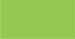
Significant result with p < 0.05 (expected direction)*.

*
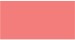
Result with trend p < 0.1 (unexpected direction)*.

*
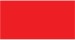
Significant result with p < 0.05 (unexpected direction)*.

### Limitations and Clinical Implications

This study may have underestimated the overall rate of progression in patients with PD due to higher attrition rates in both pathological groups. However, although the inclusion of participants who were partially present may have limited the overall significance of the group x time interaction effect, the GEE method is expected to statistically overcome this limitation (55), and including these data increased statistical power without confounding our results [as shown in our previous study (26)]. In the same study, patterns of single task data based on non-averaged biannual assessments were proven similar to averaged data. This indicates that averaging data would not have confounded our current results.

Several limitations of this study must be mentioned with particular focus on dual-task assessment, since other limitations (lack of analysis on exponential changes, high attrition rates on data, effect of averaging biannual assessments, analysis of relationship between direction of trials and lateralization effect of PD, consideration of subclinical profiles of PD such as tremor dominant and postural instability and gait disorder, lack of levodopa equivalent dose data and cut-off value for data stratification) were already discussed in our previous publication (26). Regarding dual-task assessments we acknowledge that the different tasks were not randomized leading to potential learning effects. However, this effect is likely comparable among groups (33). We did not control for factors that can modulate the capacity to cope with a concurrent cognitive load while walking, such as stress, processing speed and cognitive reserve. (27) For instance, the cognitive task Subtract depends on arithmetic skills, which are very heterogeneous between individuals (56). DTI was included in the analysis to focus on the relative impact of dual-tasking over single tasking, partially avoiding such individuals effects.

Overall, the results showed that dual-tasking did not have added value in the study of PD progression from circular gait assessments over a span of 5 years, although the results reinforce the validity of some of the gait features as progression markers of PD. This indicates that while single-task walking might be sensitive enough to the progression of PD, dual-tasking may introduce additional (error) variance to the data and may represent complex composite measures of cognitive and motor performance. There are several clinical implications related to this. Based on these results, we would argue that single-task walking might be sensitive enough to assess progression in PD, avoiding additional assessments of dual-tasking and thus reducing the burden imposed on patients and clinicians. However, in view of the sensitivity of gait-related dual tasks costs in the early stage of PD, their assessment might have added value in the identification of preclinical stages of PD.

## Ethics Statement

The Declaration of Helsinki was respected; local ethics committee approval was obtained (Medical Faculty, University Hospital of Tübingen, No. 46/2010 BO1) and all subjects provided informed written consent for participation in the study and for publication of individual, anonymized data.

## Author Contributions

MM-A developed the study and method, analyzed the results and drafted the manuscript. IK contributed to the development of the study and method, participated in the interpretation of the data and helped to draft the manuscript. SH supported the statistical analysis. TH and SN collected the data. RL participated in the coordination of the study. DB designed and coordinated the complete MODEP study and provided ideas to draft the manuscript. WM conceived and coordinated the complete MODEP study, supported the analysis and reviewed the manuscript. JD participated in the coordination of the study, in the development of the method, in the interpretation of data and in the drafting of the manuscript. All authors read and approved the final manuscript.

### Conflict of Interest Statement

McRoberts B.V. Company is the manufacturer of the DynaPort Hybrid sensor that was used in this study. RL is the founder and owner of McRoberts B.V. and was involved in the coordination of the study and in drafting the manuscript. The remaining authors declare that the research was conducted in the absence of any commercial or financial relationships that could be construed as a potential conflict of interest.

## Supplementary Material

Supplementary Table A1 Flowchart visits. Biannual visits performed by each of the participants. Complete datasets of 5 consecutive year visits were not always available in the MODEP cohort (HC: 52.0%; Early-PD: 59.1%; Mid-PD: 40.7%). This was partially due to the fact that some participants were recruited later in the MODEP study and therefore have not (yet) completed 10 visits, thus these participants may not be considered as drop-outs.

Supplementary Table B1 Mean and standard deviation values of all features. Mean ± standard deviation of all features being not log transformed, nor z-score normalized. The data is presented for the three groups (Early-PD, Mid-PD, and HC) and for the three protocols (Walk, W-Subtract, W-Mark). SD, standard deviation; VT, vertical acceleration; ML, medio-lateral acceleration; AP, anterior-posterior acceleration; Walk, single task walking in circles; W-Mark, walking in circles while marking-crosses in the paper sheet; W-Subtract, walking in circles while subtracting serial 7 digits.

Supplementary Table C1 Confounders included in the GEE analysis for the comparison Early-PD vs. HC and Mid-PD vs. HC for the three conditions: Walk, W-Subtract and W-Mark. Significant confounders that were accounted for the analysis are indicated as follows: “a” for gait speed effect, “b” for age effect and “c” for ON/OFF medication state effect. Note that the gait feature “Total duration” was not corrected for gait speed. SD, standard deviation; VT, vertical acceleration; ML, medio-lateral acceleration; AP, anterior-posterior acceleration; Walk, single task walking in circles; W-Mark, walking in circles while marking-crosses in the paper sheet; W-Subtract, walking in circles while subtracting serial 7 digits.

Supplementary Table C2 Time factor corresponding to the GEE analysis for the comparison Early-PD vs. HC and Mid-PD vs. HC and obtained for gait features under the three conditions: Walk, W-Subtract and W-Mark. Significance of results was marked with dark gray (*p* < 0.05) and light gray (*p* < 0.10). SD, standard deviation; VT, vertical acceleration; ML, medio-lateral acceleration; AP, anterior-posterior acceleration; W-Mark, walking in circles while marking-crosses in the paper sheet; W-Subtract, walking in circles while subtracting serial 7 digits.

Supplementary Table C3 Time factor corresponding to the GEE analysis for the comparison Early-PD vs. HC and Mid-PD vs. HC and obtained for DTI and S-DTI data under both dual-tasking conditions: W-Subtract and W-Mark. Significance of results was marked with dark gray (*p* < 0.05) and light gray (*p* < 0.10). SD, standard deviation; VT, vertical acceleration; ML, medio-lateral acceleration; AP, anterior-posterior acceleration; W-Mark, walking in circles while marking-crosses in the paper sheet; W-Subtract, walking in circles while subtracting serial 7 digits; DTI, dual-task-interference; S-DTI, summed dual-task-interference.

## Abbreviations

PD: Parkinson's disease
HC: Healthy control
MODEP: Modeling epidemiological data to study PD progression
BFS: Body-fixed-sensor
VT: Vertical direction
ML: Medio-lateral direction
AP: Anterior-posterior direction
MMSE: Mini Mental State Examination
SD: Standard deviation
PSD: Power spectral density
GEE: generalized estimating equations
SE: standard error
W: task of walking in circles
Mark: task of marking-crosses in the paper sheet
Subtract: task of subtracting serial 7 digits
W-Mark, dual-tasking: walking while marking-crosses on a paper sheet
W-Subtract, dual tasking: walking while subtracting serial 7 digits
DTI: dual-task-interference
S-DTI: summed dual-task-interference.
